# A comprehensive review on immune checkpoint inhibitors induced cardiotoxicity characteristics and associated factors

**DOI:** 10.1186/s40001-023-01464-1

**Published:** 2023-11-08

**Authors:** Fabrice Yves Ndjana lessomo, Oscar Onayi Mandizadza, Chishimba Mukuka, Zhi-Quan Wang

**Affiliations:** 1 Cardiology Department, Zhongnan Hospital of Wuhan University, Wuhan, China; 2 Zhejiang Chinese Medical University, Hangzhou, China; 3 Mansa Hospital, Mansa, Zambia

**Keywords:** Risk factor, Myocarditis, Immune checkpoint, Therapy, Cancer

## Abstract

Newly approved cancer drugs called ICIs have shown remarkable success in improving patient survival rates, but they also have the potential for inflammatory and immune-related side effects, including those affecting the cardiovascular system. Research has been conducted to understand the development of these toxicities and identify risk factors. This review focuses on the characteristics of ICI-induced cardiotoxicity and discusses the reported risk factors. It is important for cardio-oncologists to understand the basic concepts of these drugs to better understand how cardiotoxicities occur. It might be hard to find reports, where all patients treated with ICIs had developed cardiac toxicity, because there could be other existing and variable factors that influence the likelihood or risk of developing cardiotoxicity during treatment. Various clinical parameters have been explored as potential risk factors, and further investigation is needed through large-scale studies.

## Background

Since 2011, immune checkpoint inhibitors, hereby referred to as ICIs, have become an essential part of cancer immunotherapy, particularly with the approval of anti-CTLA-4 for advanced melanoma. These ICIs have greatly impacted the field, and since 2016, other monoclonal antibodies, such as anti-PD-1 and anti-PD-L1, have also gained acceptance in oncology therapy guidelines. These ICIs are being rapidly approved by the FDA to treat various types of cancer, greatly improving patient survival rates compared to traditional chemotherapy [1, 2]. A study involving patients with advanced lung cancer found that using pembrolizumab as a first treatment resulted in better outcomes than chemotherapy. This led to the approval of pembrolizumab by the FDA [3]. However, this great improvement added significant systemic inflammatory response potential and immune-related effects affecting diverse systems, among which the cardiovascular system has been associated with their use [4, 4]. Felice Crocetto et al. highlighted through a reliable meta-analysis that despite the benefit attached to the use of ICIs in combination with anti-VEGF (vascular endothelial growth factor) versus anti-VEGF single therapy. Patients receiving anti-VEGF therapy with ICI had a higher risk of developing cardiac and blood-related clotting disorders than those who only received anti-VEGF therapy [150]. The 2022 meta-analysis by Maobai Liu et al. showed varying incidences of cardiotoxicity among different ICI therapies. For ICI monotherapy, CTLA-4 may be associated with higher grade 3–5 cardiotoxicity than PD-1 or PD-L1 for dual therapy. The cardiotoxicity of dual ICI therapy seems to be higher than that of chemotherapy or targeted therapy [151].

Because most patients did not undergo systematic and routine cardiovascular status monitoring, cardiac adverse events seemed under-reported in the literature. Initial investigations revealed that mice deficient in PD-1 developed dilated cardiomyopathy and severe myocarditis [6–8]. According to a 2016 pharmacovigilance analysis report, approximately 0.09% of patients treated with nivolumab, another PD1 ICI, had developed myocarditis; when combined with ipilimumab, the incidence of myocarditis was approximately 0.27%. Other cardiotoxic effects reported include pericarditis, pericardial effusion, cardiomyopathy, and new arrhythmias [9–11]. The incidence of ICI-induced cardiac toxicity is relatively low, ranging from less than 1% to approximately 18%, depending on the specific ICI and patient population studied. However, it can be a severe and potentially life-threatening complication.

Current investigations focus on how ICIs cause heart-related side effects and the identification of risk factors. The currently established risk factors associated with ICI-induced cardiac toxicity include pre-existing cardiovascular disease, concomitant medications, ICI type and dosage, prior exposure to cardiotoxic therapies, autoimmune diseases, age, and sex, most of which are considered traditional cardiovascular risk factors. Nevertheless, several other factors are sparse in the literature. It would still be interesting to look closely at them, as close monitoring is needed to detect and manage any potential cardiac toxicity promptly. This review focuses on the characteristics of ICI-induced cardiotoxicity and discusses the probable ICI-induced cardiotoxicity risk factors from the available literature. This concise and focused review might help design cardiotoxicity risk stratification in the setting of ICI-induced cardiotoxicity.

### Overview of ICIs

#### Definition

ICIs block the function or effect of immune checkpoints, which are immune components expressed on the cell surface of T lymphocyte cells. There are two types of ICIs: stimulatory checkpoints that potentiate immune cell action and activation (TNF, CD27, CD40) and inhibitory checkpoints that downregulate the immune response (CTLA-4, PD1, IDO, KIR, LAG3) [12–15]. The inhibitory type is targeted by ICI therapy. In 1968, researchers discovered that lymphocyte cells from cancer patients could react against cancer cells in vitro. These inhibitory checkpoints were identified in 1995 by Ph.D. Jim Allison [16–20]. Jim Allison discovered that the protein CTLA-4 controls T-cell activation and recruitment [21–23]. PD1 is another immune checkpoint receptor that intervenes at two levels: differentiation of immature precursor T cells into effector and memory T-cell populations and activation or reactivation of circulating or resident effector and memory T-cell subsets. Blocking PD1 improves antitumour CD8 + T-cell cytotoxic capacity by reducing the tumor-suppressive impact of PDL1 and PDL2 produced by neoplastic cells [22–30].

#### Classification of ICI drugs

ICIs are classified based on the type of immune checkpoint. FDA-approved immune checkpoint blockade drugs have three main subclasses that share almost the same indications and adverse effects but have distinct pharmacodynamic and pharmacokinetic properties [27–32]. In addition, new generations of ICI drugs, including LYMPHOCYTE ACTIVATION GENE-3 INHIBITORS (LAG-3 INHIBITORS), are still being investigated for potential clinical use. Table 1 summarizes the ICI drug classification, FDA-approved year, and indications [30–47].

**Table 1:** Classification of ICIs

Generic name and date of FDA approbation	Subclass	Mechanism of action	indications
Ipilumab	CTLA-4 ICI	Ipilumab enables the patient’s T cells to attack a broader range of antigens rather than inducing an increase in T cells. Ipilimumab binding to CTLA-4 blocks the inhibitory signal, thus, allowing CTLs to kill cancer cells [31–33]	Melanomas Colorectal carcinoma Oesophageal cancer Hepatocellular carcinoma Non-small cell lung cancers Renal cell cancers [38]
nivolumab	PD1 ICI	relieves immune cells from pathological immune suppression and allows them to recognize and combat tumor cells by inhibiting PD-1 activity [43]	Esophageal squamous cell carcinoma (ESCC) Classical Hodgkin Lymphoma Hepatocellular carcinoma Colorectal cancer Urothelial carcinoma Small cell lung carcinoma, metastasis Pleural mesothelial Renal cell carcinoma [40–42]
Pembrolizumab [44]	PD1 ICI	Like Nivolumab	Melanoma, non-small cell lung cancer, head and neck squamous cell cancer, classical Hodgkin lymphoma, urothelial carcinoma, GIT cancers cervical cancer hepatocellular carcinoma and Merkel cell carcinoma
Atezolizumab	PDL1 ICI	increase the number of proliferating CD8 + T cells by inducing increases in IL-18, IFN, and CXCL11 and a temporary decrease in IL-6 [45, 46]. by inhibiting PD-L1, thus increasing T-cell-mediated immunity against tumors	Safe and efficacious in a wide range of solid tumors and hematologic malignancies metastatic NSCLC unresponsive to platinum-containing chemotherapy extensive-stage small-cell lung cancer [20, 47]

### Immune checkpoint inhibitors and cardiotoxicity

#### *Mechanism of immune checkpoint inhibitor therapy-induced cardiotoxicity (*Fig. 1*)*

**Fig. 1:**
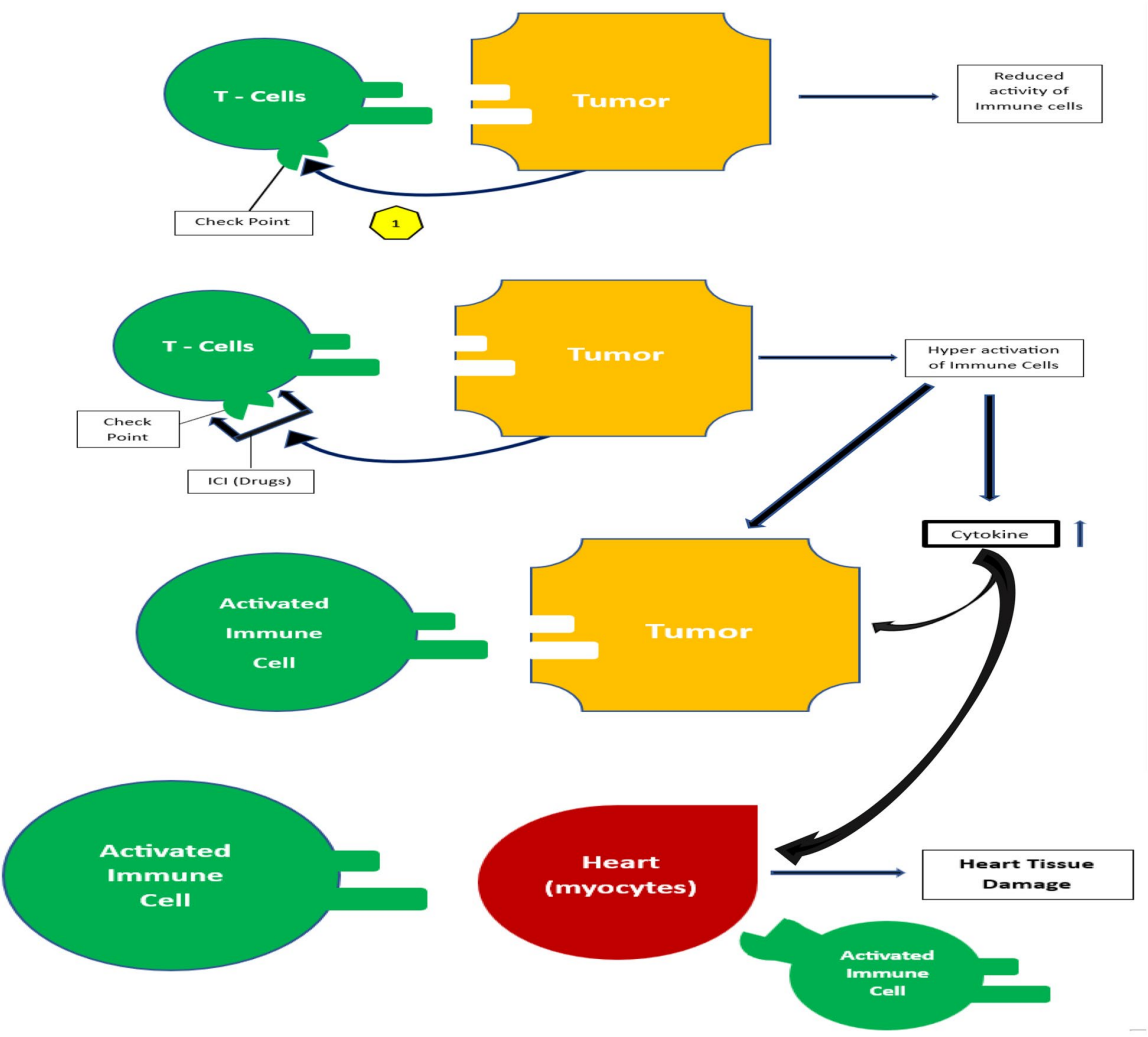
Simplified mechanism of ICI-induced cardiotoxicity

According to the 2019 World Health Organization's global database analysis, on 12 455 401 ICI drug case safety reports, patients who received ICIs had an 11-fold higher likelihood of reporting myocarditis than those who did not receive ICIs [48, 49]. Furthermore, based on the Joe-Elie Salem et al. 2018 study, ICI treatment has related to other inflammatory cardiovascular side effects, including pericardial diseases and vasculitis, with a higher occurrence of temporal arteritis. Reports have also linked ICIs with noninflammatory cardiovascular toxicity, such as Takotsubo-like syndrome. However, it was difficult to solely attribute these effects to pembrolizumab, because patients may have received other medications known for their chronic cardiotoxic properties, such as trastuzumab [50, 51].

Other cases of noninflammatory cardiovascular toxicity have been reported, including symptom-free, noninflammatory left ventricular dysfunction, myocardial infarction, and coronary vasospasm. [52–54]. It is now recognized that arrhythmias can indicate cardiotoxicity in patients undergoing ICI treatment. However, arrhythmias are common among individuals with cancer and often occur alongside other immune-related adverse events. Examples of these events include acute thyrotoxicosis observed in ICI-mediated thyroiditis [48, 54]. In case reports, ICI-associated third-degree atrioventricular block and conduction disease were often attributed to conduction system disturbances secondary to myocarditis [7, 55, 56].

The mechanisms underlying cardiovascular irAEs are not well-understood. For example, ICI-induced myocarditis is characterized by infiltration of macrophages and T lymphocytes in the myocardium, leading to cell damage and death [57]. ICI-associated myocarditis involves the infiltration of CD4 + and CD8 + T cells and CD68 + macrophages into the myocardium and conduction system. B cells are in short supply. Understanding cellular tolerance and triggers for T-cell infiltration is crucial. A case series study found consistent immune aberrancy in patients with fulminant myocarditis, mainly involving striated muscle and tumours [58]. Multiple tissue types showed robust T-cell infiltration, activation, and clonal expansion with evidence of shared high-frequency T-cell receptors. The probable mechanistic hypotheses that were then proposed included the following:

1. T cells targeted an antigen that was simultaneously present across tumour, skeletal muscle, and heart tissues; this could be supported by the finding of high levels of muscle-specific antigens (desmin and troponin) in both patients' tumours,
2. The same T-cell receptor targeted a tumour antigen and a different but homologous one sharing the same spatial conformation with a specific muscle antigen
3. Clonal, high-frequency T-cell receptor sequences across tumour and muscle samples could be misleading, and distinct T-cell receptor specificities target dissimilar antigens

Although extensive viral profiling revealed no clear etiology, it was hypothesized that subclinical viral infection could have generated T-cell targets. However, that study failed to prove the existence of common HLA alleles among patients. This led to a nonplausible HLA/drug hypersensitivity association theory. Therefore, the underlying causes of T-cell reactivity to myocardial and other striated muscle tissue are unknown and are certainly not universal across patients.

Currently, the available theories in research mainly focus on understanding the early mechanism of immune-related reactions induced by ICIs [59, 60]. Studies have shown that immune checkpoint inhibition can lead to myocarditis. In mouse models of T-cell-mediated myocarditis, PD-1 plays a significant role in protecting against inflammation and myocyte damage [61]. However, there is a notable amount of PDL1 expression in the heart muscle of individuals with ICI-induced fulminant myocarditis, which aligns with the increased level of this marker found in preclinical studies and mouse models. This suggests that PDL1 may play a protective role in preventing heart damage [62–64]. PDL1 upregulation has been linked to a cytokine-induced mechanism that protects the heart and is now disrupted by ICI blockade [64]. Activated immune cells can infiltrate normal muscle cells, including those in the heart, due to similarities between tumors and body muscle antigens. This can cause cardiotoxicity via complex mechanisms, including direct cell killing and increased pro-inflammatory cytokine levels. This is considered “bystander effect” heart damage. Approval for cancer immunotherapy has been granted to cytokines, such as IL-2, which have anticancer properties; however, they are also rare causes of myocarditis, occurring in 1.5% of 652 cases [65, 66]. High levels of cytokines such as IL-2, IFN-y, TNF, IL-1, and IL-6 have been linked to myocarditis. This condition causes inflammation and damage to the heart muscles. Studies have shown that 6 out of 8 patients with high IL-2 levels had myocarditis. Similar results were observed in autopsies of subjects with elevated levels of other cytokines [67–69]. Activated lymphocytes can indirectly affect heart tissue through the release of interferon-alpha and interleukin-2. CD8 + T cells produce interferon-gamma and TNF-alpha when activated by Th1 cells. Blocking immune checkpoints could lead to potential tumor destruction but also harm heart tissue due to increased cytokine levels.

ICI therapy can affect immune function in immune-privileged organs, such as the heart, which has few T cells and defensive mechanisms against T-cell attacks. The myocardium secretes IFN-γ and upregulates PD-L1 to reduce T-cell damage and prevent the growth of T helper cells, causing myocarditis. [136–138]. The illustration below summarizes the possible patterns that could be a rationale for cardiac damage after immune checkpoint blockade in the oncological management setting.

#### Spectrum of ICI-induced cardiotoxicity

The heart is vulnerable to inflammation and damage due to its dense vascularity, which can cause arrhythmias. Cardiovascular toxicities from ICI therapy are becoming more common and can result in myocarditis, pericardial illnesses, vasculitis, Takotsubo syndrome, conduction problems and unstable atherosclerotic lesions [70]. A 2014–2019 pharmacovigilance study by Chenxin et al. also reported that the spectrum of ICI-induced cardiotoxicity differed between ICI drug types and regimens but shared some similarities. The top five cardiac adverse events recorded in the database were dyspnea (21%), myocarditis (5.16%), atrial fibrillation (4%), cardiac failure (4%), and pericardial effusion (3.5%). In a comprehensive analysis by Hu et al. comprising 22 clinical trials evaluating PD-1 and PD-L1 inhibitors for lung cancer, the frequency of myocarditis was 0.5%. However, the frequency of other cardiovascular adversities, such as pericardial tamponade, myocardial infarction, stroke, cardiac failure, and cardiorespiratory arrest, varied between 0.7% and 2.0%.

**Fig. 2:**
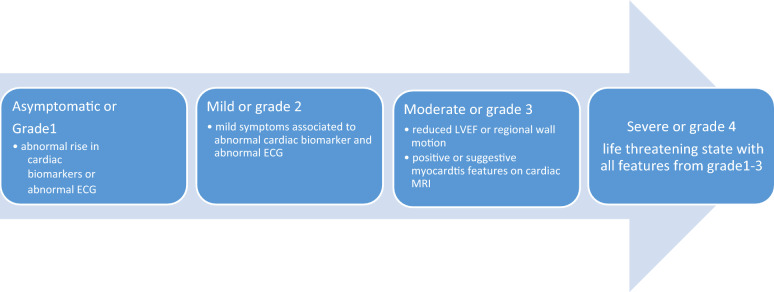
ICI-induced myocarditis severity grading

Myocarditis is an inflammatory disease of the myocardium caused by several factors, such as viral infections, toxins, hypersensitivity reactions, and autoimmune disease. Autoimmunity has strongly been implicated in the etiology and progression of myocarditis. Many murine and clinical studies have reported on the plausibility of myocarditis onset in association with ICI use [71, 72]. The number of myocarditis reports is gradually growing, consistent with the increasing trend of ICI-induced cardiac damage. The prevalence of myocarditis and reporting is expected to continue to rise over time. Anti-PD-1, especially nivolumab, was hypothesized to have a more robust signal value in myocarditis [73]. Myocarditis is known to occur in the acute stage of ICI treatment. A past study found that the median time to onset from initiation of therapy to occurrence of symptoms was approximately 30 days [74]. Therefore, ICI-induced myocarditis seemed to be early onset cardiac toxicity in single and combination ICI therapy settings, although most cases were observed with a single therapy [74–76]. The clinical tableau can differ from asymptomatic cardiac biomarkers to severe decompensation, with the propensity for end-organ damage. The American Society of Clinical Oncology (ASCO) clinical practice guidelines have proposed a classification and grading system for myocarditis as shown below [77–79] (see Fig. 2). When myocarditis is suspected during ICI treatment, a thorough workup is recommended regardless of severity. This includes cardiac markers, electrocardiography, thoracic radiography, echocardiography, and referral to a cardiologist for further testing, such as cardiac magnetic resonance imaging, coronary angiography, and endomyocardial biopsy [77]. Medical practitioners may not have complete knowledge about myocarditis, leading to uncertainty about when to suspect or consider the condition. Increased troponin levels without symptoms may indicate mild myocarditis, but other factors can also cause troponin elevation [80]. Consequently, another classification of myocarditis was suggested as a three-level categorization: definite myocarditis, probable myocarditis, and possible myocarditis [81]. Cardiac MRI is still the best and least invasive way to diagnose myocarditis, even though endomyocardial biopsy is the gold standard [80]. Overall, it would be ideal for patients with suspected myocarditis to go through most diagnostic studies listed above until more definitive evidence becomes available.

Immune checkpoint inhibitors (ICIs) may increase cancer patients' risk of pericardial disease, which could lead to higher mortality rates. ICI-induced pericardial disease is rare and has varying incidences and presentations, which may cause delayed diagnosis and treatment. A recent pharmacovigilance study recorded that the incidence of ICI-induced pericardial disease, including pericarditis and pericardial effusion, was estimated at 0.36% [82]. Pericardial disease can occur alongside myocarditis or on its own, leading to pericardial effusion and cardiac tamponade. Previous research has identified several cases of pericardial disease associated with immune checkpoint inhibitor (ICI) treatment, with nivolumab being the most common. Most cases showed symptoms of tamponade, while some had effusive–constrictive physiology. Pericarditis typically developed 6 weeks and 11 months after starting ICI treatment, with one exception occurring 4 days later. Pericardiocentesis was the main treatment option, performed in five cases. Analysis of the pericardial fluid showed the presence of white blood cells, mostly lymphocytes, and no signs of cancer [83–87]. Pericardial disease from ICI can cause chest pain, difficulty breathing, and respiratory failure. Diagnosis involves identifying pericarditis, which can lead to effusion and tamponade [88, 89]. CT scans show effusion and thickening, while MRIs show inflammation. Coexisting myocarditis may raise troponin levels [80, 88].

Cancer treatment-induced arrhythmia (CTIA) is a potential side effect that may occur during chemotherapy. It can result in diverse types of irregular heart rhythms, including fast and slow heartbeats, which may lead to a complete heart block. Patients receiving immune checkpoint inhibitors have reported several cases of CTIA [90, 91]. In a retrospective study, 268 patients who underwent immune checkpoint inhibitor therapy were examined, and it was discovered that only 1.5% of them had a clinically significant arrhythmia within 6 months. The study also found that patients who had a previous diagnosis of atrial fibrillation were more likely to experience relapse while on immune checkpoint inhibitor therapy. The conclusion of the study was that immune checkpoint inhibitors are generally well-tolerated and safe regarding arrhythmias. A different study, which used a different database, also found similar results, but it highlighted that certain factors, such as thyrotoxicosis, may contribute to the development of atrial fibrillation [91, 92]. There is a hypothesis that drug-induced arrhythmias caused by immune checkpoint inhibitors (ICIs) tend to occur shortly after starting ICI treatment. This hypothesis applies to all types of ICI regimens and suggests that arrhythmias typically develop within 1 month of starting ICI treatment [92, 93]. Diagnosing ICI-associated arrhythmia and myocarditis lacks clear criteria. Abnormalities in ECGs and echocardiograms can serve as indicators. No established biomarkers exist for predicting outcomes. Clinicians must describe and evaluate adverse drug events [94, 95]. A recent study showed that starting ICI therapy can increase the risk of adverse arrhythmic events, with a 26% mortality rate associated with CTIA. It is crucial to be aware of this risk and not overlook CTIA.

Athero-cardiovascular toxicities include large vasculitis, coronary artery disease, thromboembolic events, and even myocardial infarction. Evidence suggests that ICIs significantly contribute to atherogenic T-cell activation, atherosclerosis development and coronary function regulation. PD1-depleted mice exhibited increased development of atherosclerotic lesions compared to controls. T cells have been found to play a pivotal role in advancing atherosclerosis towards more advanced, clinically unfavorable lesions. In addition, they have been directly implicated in plaque rupture and subsequent development of acute cardiovascular events [48, 53, 96]. ICIs activate proatherogenic T-cell immunity, increasing interferon and tumor necrosis factor production and raising the risk of coronary thrombosis [97]. Recently, some cases of retrosternal chest pain that did not meet the myocarditis diagnostic criteria and were more like acute coronary syndrome angina pain have been identified among patients taking ICI medication [98, 99]. According to a pharmacovigilance study, this type of cardiotoxicity accounted for 0.53% of all case safety reports. It was reported to be more common than ventricular arrhythmia (0.07%) and cardiac death (0.43%) [49]. In the JOCARDITE registry study (*n* = 474), only 55.1% of ICI-myocarditis patients underwent coronary angiography, and 22.6% had concomitant CAD. In a recent pharmacovigilance study, CAD represented one-third of all cases [100]. It is possible that many patients diagnosed with ICI-induced myocarditis have coronary artery disease, leading to a lower number of reported cases of CAD in studies. Various imaging studies have shown that ICIs can contribute to inflammation of large blood vessels and the formation of atherosclerotic plaques, not limited to CAD [101–103]. One could argue that atherosclerosis is a slow process that takes a long time before its symptoms or complications become apparent. In patients, the presence of conventional cardiology risk factors may have contributed to the development of ICI-induced CAD and vasculitis. However, currently, traditional risk factors such as age, obesity, and smoking are still considered independent of the risk of ICI-induced cardiac toxicity [104]. CT scans can predict the risk of atherosclerosis in coronary arteries. More research is needed to determine the risk of mortality in ICI-induced CAD [105]. Coronary angiography could become a routine investigation for cancer patients on ICI therapy.

We have presented the major type of cardiac toxicity occurring with ICI medication. However, the list may not be exhaustive. Cases of Takotsubo were also reported and confirmed by negative endomyocardial biopsy [106–108]. Takotsubo cardiomyopathy is linked to advanced malignancies and potential triggers, such as emotional disturbances, cancer treatment, and chemotherapy. The pathophysiology is unclear, although hypotheses include coronary vasospasm, microvascular dysfunction, and excessive stress response. Facts from rodent model studies have suggested that inflammation plays a crucial role in Takotsubo cardiomyopathy [106, 108].

Hypertension is also a reported side effect of ICI use. Nevertheless, a recent meta-analysis did not find a significant increase in the short-term risk of hypertension among patients treated with these drugs [109]. ICI-induced hypertension and atherosclerosis are likely related. A case of pulmonary artery hypertension was seen in a non-smoking African American woman with lung cancer who was treated with chemotherapy and an ICI drug. She also developed insulin-dependent diabetes, hypothyroidism, and adrenal insufficiency [110]. Therefore, the greater propensity of ICI drugs to lead to autoimmune conditions puts every patient taking those drugs on the higher watch for autoimmune-related pulmonary artery hypertension (PAH type 1). Finally, heart failure is a long-term complication of both cancer and cancer treatment-induced cardiac toxicities and has been consistently reported in patients with ICI-induced myocarditis.

### Parameters correlating with increased risk of ICI-induced cardiac toxicities

Identifying literature reports, where all patients who received ICI drugs developed cardiac toxicity is challenging due to other risk factors that may affect the outcome. Various parameters that vary from patient to patient have been analysed to determine if they could be considered risk factors. These parameters have been classified into three types, which are outlined in Table 2.

**Table 2:** Parameters associated with risk ICI-induced cardiac toxicity

General	Clinical	Para-clinical
Ethnicity/race	History of chronic disease	Inflammation biomarkers
Age	CVD Hx and risk factors	Cardiac biomarkers
Sex	Heart rate	genetic biomarkers
financial status	Fever	microRNAs

#### General parameters

Ethnicity or race is a crucial factor with higher variability that can impact diagnosis, treatment, and prognosis. For instance, the administration of beta-blockers and ACEis in the hypertensive management approach, atherosclerotic cardiovascular disease score risk (ASCVD), and EGFR estimation depend on the patient’s ethnicity [111–113]. In addition, an individual's self-identified race is a reliable indicator of overall health and longevity [112]. Therefore, the rarity of studies addressing the impact of ethnicity on the risk of ICI-induced cardiac toxicity would be paradoxical. Zakary et al. 2022, a retrospective study, found that from 468 Caucasians exposed to ICI therapy, only 19 developed cardiotoxicities, while for 57 African American natives, only 7 developed the outcome. Therefore, the difference in cardiac toxicity percentage was significant (*P* < 0.05). This made the African American ethnicity or race more susceptible to the onset of cardiac events when exposed to ICI [111]. However, although significant, this result would give clues but not answer the question. There is still a pending for some high-level perspective and meta-analysis studies to conclude the matter.

In medicine, gender is a crucial factor to consider. For example, if lung cancer is more prevalent in females, it could be because more women are exposed to ICIs than men, leading to higher rates of cardiac toxicity among women. However, when examining the range of cancer types that can be treated with ICIs, it is important to look at studies that include all types of cancers to determine if there is a consistent gender pattern of association with the risk of cardiac toxicity. Zakary et al. found that a significantly higher percentage of women experienced cardiac events compared to men. However, Maria et al.'s study did not find any significant gender association with the risk of ICI-induced cardiotoxicity [114]. However, this discrepancy is not without reason. We would like to recall that male sex is generally a risk factor for cardiovascular pathology. In contrast, females have a greater tendency to have autoimmune disease and lung cancer than men [115, 116]. Because the autoimmune reaction is also encountered with ICIs, it appears hard to predict which gender carries a greater risk for cardiac toxicity. While we wait for proper specification, patients from all genders would still need equal attention during screening, monitoring, and follow-up after initiation of ICI.

Age would be a constant parameter. The spontaneous or natural risk for cardiovascular disease increases with age; on the other hand, the severity of cancer and the effectiveness of response to therapy are all influenced by the age of the patients [117–119]. According to the universal theory, patients who are more advanced in age are at a higher risk of developing comorbidities, including when undergoing ICI therapy. Studies have shown that even myocarditis, which typically affects younger patients, can occur with ICI treatment, and the risk increases with age. In the Maria et al. study, the case group had a mean age of 65 years compared to 59 years in the control group, and this difference was statistically significant [114]. Consequently, when evaluating patient ICI-induced cardiotoxicity risk, cardio-oncologists could consider the elderly as a higher risk cohort and accentuate their therapeutic surveillance.

Some patients may be unable to receive adequate cancer treatment due to the excessive cost, leading to severe complications. Therefore, healthcare providers must consider a patient's financial status when reviewing their medical history, as it can influence their treatment plan. Each patient's economic situation is unique and should be given proper consideration. At present, guidelines do not prioritize financial status when figuring out care, diagnosis, and follow-ups. However, this should be re-evaluated [120]. While health is often said to be priceless, the goal of medicine should be to supply healthcare for everyone.

#### Clinical parameters

Studies addressing the association between cardiovascular risk factors and the risk of ICI-induced cardiac toxicity, for the majority, assessed smoking, BMI, hypertension, and diabetes mellitus. Zachary et al. reported a significant association between smoking and the risk of ICI-induced cardiotoxicity.

A study found that among the 354 smokers analysed, there was a significant incidence of cardiac toxicity (4.212 [1.289, 13.763] *P* < 0.05). Smoking is a well-known risk factor for lung cancer and can negatively affect various treatments for the disease. Smoking increases the presence of PD-L1, impairs the body's ability to fight inflammation, and allows cancer cells to go undetected by the immune system. It also causes inflammation in the tumor microenvironment, promoting tumor growth and exhausting T cells. [121, 122]. It was also hypothesized that smoking history in patients suffering from NSCLC significantly determines their response to ICI treatment. It seemed to depict a trend towards improvement [123]. Therefore, the action of ICI is potentiated by the presence of a smoking history; tobacco components act in synergy with ICI drugs, which could increase the risk of immune-related adverse events, such as myocarditis and pneumonitis. As a result, it could be thought that smokers may be associated with a double the risk for cardiac events, one from the tendency of smoking to induce atherosclerosis and another from the activation of T cells.

Zachary et al. also reported a nonsignificantly increased risk of developing cardiac events with BMI, hypertension, or diabetes mellitus type II; a similar observation trend could also be found in the Maria et al. report. Whether ICI can provoke hypertension has been discussed in the earlier section. However, the occurrence of cardiac events among hypertensive patients taking ICIs appears independent of the ICI drug effect. However, patients with hypertension have a greater risk for other cardiac diseases, such as ischemic heart disease and arrhythmia, whose histories were significantly associated with the onset of cardiac events in a multivariate analysis [114]. Therefore, cardiovascular disease history would be a significant predisposing factor. However, the mechanism of this association still needs to be further elucidated.

A recent study found that people with both type 2 diabetes and cancer may have worse outcomes when receiving immune checkpoint inhibitor therapy. The study involved 1395 patients with advanced solid tumours who received this therapy between 2014 and 2020. The analysis showed that patients taking diabetic medication had shorter overall survival and progression-free survival than those who were not taking the medication or likely did not have diabetes [124]. Although incomplete, because the study did not specify cardiotoxicity development among those patients. However, at least it appears clear that a history of diabetes may affect the outcome of ICI. Nevertheless, possible side effects result from ICI use, in people with diabetes present a 2–4 times greater risk for cardiac-related adverse events [125, 126]. Further studies need to be deployed to evaluate the effect of DM on the risk of ICI-induced cardiotoxicity. Nevertheless, diabetes mellitus should always be considered when estimating the risk of any cardiovascular pathologies, including cancer treatment-induced pathologies.

Obesity is a significant cardiovascular risk factor, and some studies have suggested that there is reduced efficacy of cancer treatment among obese patients for some types of treatments, particularly chemotherapy because of underdosing by providers. Nonetheless, recent research underscores the obesity paradox, which points to a correlation between increased body mass index (BMI) and favorable results in cases treated with immune checkpoint inhibitor (ICI) therapies [126, 128]. However, little data on its correlation with the risk for ICI-induced cardiotoxicity is discernible in the literature, but obesity increases exhausted T cells, with mice showing higher PD-1 expression. CD4 + and CD8 + T cells displayed reduced proliferation and cytokine production when stimulated ex vivo in mice and humans [54]. Obese melanoma patients over 60 show increased PD-1 and other exhaustion markers and lower T-cell proliferation [129]. Obese individuals with important levels of the hormone leptin may have increased expression of PD-1, a protein found in tumor-infiltrating lymphocytes, which can lead to worse cancer outcomes. Leptin triggers STAT3 activation, leading to increased transcription and translation of PD-1. This can ultimately lead to reduced T-cell function and proliferation, negatively impacting cancer treatment. However, high BMI may have a positive effect on immunotherapy effectiveness, but administering immunotherapy to obese patients may increase immune-related adverse effects [130]. Obese mice in the non-ICI group had chronic inflammation. Overweight patients have a higher chance of immune-related adverse events. However, the effects are not severe. Treatment effectiveness increases the risk of adverse events, and weight loss does not have significant benefits [131]. Zachary et al. found that obesity was significantly associated with mortality in ICI settings.

Reduced glomerular filtration, as seen in renal failure, has been significantly linked to the onset of immune-related renal adverse events [132]. Although the Maria et al. study found a nonsignificant association between a history of chronic renal failure and risk for cardiac events in patients taking ICI medications, it could still be possible to identify an indirect link between pretreatment reduced glomerular filtration rate and risk for cardiac toxicity. Pretreatment reduced GFR, leading to renal adverse events, which, in turn, affect the heart in numerous ways. Reduced ejection fraction is widely accepted as a cardiovascular pathology risk factor [133]. Therefore, renal patients undergoing ICI therapy courses should be proactively managed and followed up concerning cardiovascular toxicities.

Treatment-related anemia can raise heart rate and increase the risk of heart failure and myocardial ischemia in patients with high heart rates before treatment. Further research is needed to confirm this theory. Fever can affect the immune response and tumor microenvironment, but mild hyperthermia combined with immune checkpoint inhibitors has shown promise in preclinical studies and appears safe [134, 135]. If the action of ICIs is promoted or increased, the propensity for cardiac toxicity also increases; thus, baseline fever, which might also indicate an infection in an ICI candidate, could be considered a predisposing factor for cardiotoxicity, and this remains to be demonstrated with future studies.

#### Paraclinical

This section explores nonclinical parameters such as inflammatory or indices, cardiac biomarkers, genetic cancer biomarkers, imaging, and therapy-related parameters to show their relationship with cardiac toxicity in an ICI setting.

Inflammation is the cornerstone mechanism by which ICI achieves its efficacy and is also a cancer-related effect [102]. Inflammation is an immune reaction. Therefore, amplifying the immune response with ICIs equals amplifying inflammation, which may lead to organ damage. No wonder most guidelines recommend using glucocorticoid or immunosuppressant therapy to manage ICI-induced toxicities, including cardiac ones [136]. As shown in previous reports, systemic inflammatory tools and indices could also be relevant for risk stratification concerning ICI-induced cardiac toxicity [105]. For instance, the platelet-to-albumin ratio (PAR), neutrophil-to-lymphocyte ratio (NLR), platelet-to-lymphocyte ratio (PLR), lymphocyte-to-monocyte ratio (LMR), and C-reactive protein/albumin ratio (CAR), which are inflammatory indexes with prechemotherapeutic value, have been found to carry significant prognostic value in evaluating outcomes after therapy in pancreatic tumors [137–140]. Predictors that increase the risk of complications have been studied, but not in relation to ICI-induced cardiac toxicity. Immune biomarkers related to B and T cells can help assess risk. Recent studies have shown that decreased clonality and increased TCR diversity after treatment with a CTLA4-blocking antibody can lead to irAEs. CD8 + T-cell clone expansion in ICI-treated patients is also correlated with irAEs [141]. Autoantibodies can show the impact of immune checkpoint inhibitors on T and B cells, but their role in autoimmune disorders is unclear. Detecting activated T cells early on can help with patient care, and autoantibodies can predict if a patient is likely to experience immune-related adverse events. However, accurately characterizing these autoantibodies remains unresolved [142, 143].

Cardiac natriuretic peptides and troponins are useful markers in identifying patients who may be at risk of cardiotoxicity. Recent developments have made it possible to use these markers to diagnose early cardiotoxicity and predict late-onset cardiotoxicity. The current guidelines on cardio-oncology support their crucial role in detecting cardiotoxicity caused by cancer therapy [144]. The significance of monitoring troponin levels to detect cardiotoxicity has primarily been proven through investigations of individuals undergoing chemotherapy for cancer, particularly those receiving anthracyclines. A prolonged increase in troponin I levels is positively correlated with a greater degree of left ventricular (LV) dysfunction and a heightened likelihood of cardiac events compared to temporary fluctuations in troponin levels. BNP and NT-proBNP are crucial biomarkers for pressure overload, myocardial stretch, and cardiotoxicity detection. BNP can detect acute cardiotoxicity within 24 h of anthracycline chemotherapy and has been found to be useful in screening for HF in patients with dyspnea on cancer therapy. A study measured natriuretic peptide in a cohort of 600 oncologic patients, and high NT-proBNP levels indicated higher mortality risk: hazard ratio 1.54 (95% CI 1.24–1.90, *p* < 0.001) with a 67% 25-month survival rate compared to 49% for normal NT-proBNP levels [145, 146]. Therefore, measuring these biomarkers would help identify patients who could develop cardiotoxicity but may also help determine the degree of cardiac dysfunction [145, 146].

Some genetic markers, such as IL7 gene variants, may increase the risk of heart damage from immune checkpoint therapy. Certain biomarkers in lung cancer, such as epidermal growth factor and anaplastic lymphoma kinase, could reduce ICI treatment effectiveness. Tumors driven by the KRAS gene depicted good outcomes with ICI therapy [146, 147]. These observations provide evidence that genetic parameters can help predict therapy-induced toxicity. Further research is needed to determine which genetic parameters are relevant for immunotherapy-induced cardiac events. Cancer biomarkers such as CEA, NSE, CA125, and SCC are useful for monitoring treatment effectiveness and tumor severity. They may also have a potential link to ICI-induced heart damage. If these findings become available, they would significantly advance future cardio-oncology guidelines… [148, 149]

Past research has found several microRNA molecules that could potentially be biomarkers for heart toxicity. MicroRNA146a, miRNA 140-5p, and miRNA-377 have shown connections to cell death and mortality in animal models of doxorubicin-induced heart toxicity. However, further research is needed to understand how these molecules function, assess their variability, and confirm their usefulness in clinical applications for immune checkpoint inhibitor therapy.

### Discussion and future perspectives

Cardiac toxicity from ICI treatment is a concern in cancer management. The low incidence suggests that certain factors may make individuals more susceptible. Cardio-oncologists need to understand the mechanisms and watch for any parameters that may lead to toxicity. The main mechanism is an exaggerated T-cell response in myocardial cells, with myocarditis being the most common type. Ethnicity, age, gender, financial status, history of chronic disease, inflammation biomarkers, CVD history and risk factors, heart rate, cardiac biomarkers, genetic biomarkers, fever, and microRNAs have all been associated with an increased risk for ICI-induced cardiac toxicity. However, these observations come from nonrandomized studies, and the strength of association was quite weak. Additionally, it was rare to find a parameter in isolation, as multiple parameters often interact with one another. Dual or combination of ICI therapy and non ICI therapy may also affect the risk of ICI induced cardiac toxicity [150, 151]. Creating indexes and scoring that consider multiple factors could help assess the risk of cardiotoxicity caused by ICIs. Future research needs to focus on finding predictors of severe cardiac toxicity and improving the response to high-dose corticosteroids.

A study conducted by Biagio Barone and his team in 2023 has revealed a correlation between urothelial cancer and cardiovascular disease [152]. The study suggests that there are overlapping pathways in the development of both conditions, meaning that patients with urothelial cancer already have a higher incidence of cardiovascular disease at baseline. This baseline risk is further increased in patients undergoing ICI therapy, who are even more susceptible to cardiotoxic medications. Therefore, caution must be exercised when administering cardiotoxic medication to this subset of patients. Notwithstanding, the potential for ICI-induced cardiac toxicity should not be overlooked, as it can lead to some long term bad outcomes such as the development of serious conditions such as bladder cancer.

## Conclusion

This review emphasizes the importance of the early detection, prediction, and management of ICI-induced cardiac toxicity. Factors not linked to the heart or immunotherapy increase the risk of cardiac events in ICI therapy. The parameters should be thoroughly investigated in large multi-ethnic, multicentre prospective cohorts and experimental studies.

## Data Availability

All the data generated are available from the corresponding author upon reasonable request.

## Abbreviations

ICI: Immune checkpoint inhibitor
PD1: Program cell death receptor 1
PDL1: Program cell death receptor ligand 1
